# The effectiveness and cost-effectiveness of strength and balance Exergames to reduce falls risk for people aged 55 years and older in UK assisted living facilities: a multi-centre, cluster randomised controlled trial

**DOI:** 10.1186/s12916-019-1278-9

**Published:** 2019-02-28

**Authors:** Emma K. Stanmore, Alexandra Mavroeidi, Lex D. de Jong, Dawn A. Skelton, Chris J. Sutton, Valerio Benedetto, Luke A. Munford, Wytske Meekes, Vicky Bell, Chris Todd

**Affiliations:** 1 Division of Nursing, Midwifery and Social Work, School of Health Sciences, Jean McFarlane Building, University of Manchester, Oxford Road, Manchester, M13 9PL UK; 2 Manchester Academic Health Science Centre, Core Technology Facility, The University of Manchester, 46 Grafton Street, Manchester, M13 9NT UK; 3 Manchester University NHS Foundation Trust, Manchester, UK; 4 School of Health and Life Sciences, Centre for Living, Glasgow Caledonian University, Govan Mbeki Building, Cowcaddens Road, Glasgow, G4 0BA UK; 5 School of Physiotherapy and Exercise Science, Faculty of Health Sciences, Curtin University, Perth, Australia; 6 Centre for Biostatistics, Division of Population Health, Health Services Research & Primary Care, School of Health Sciences, Jean McFarlane Building, University of Manchester, Oxford Road, Manchester, M13 9PL UK; 7 Lancashire Clinical Trials Unit and Research Support Team, Faculty of Health and Wellbeing, Brook Building, University of Central Lancashire, Preston, PR1 2HE UK; 8 Manchester Centre for Health Economics, School of Health Sciences, Jean McFarlane Building, University of Manchester, Oxford Road, Manchester, M13 9PL UK; 9 Tranzo, Tilburg School of Social and Behavioural Science, University of Tilburg, 5000 LE Tilburg, The Netherlands; 10 School of Psychological Sciences & Health University of Strathclyde Graham Hills Building, Glasgow, G1 1QE UK

## Abstract

**Background:**

Falls are the leading cause of fatal and non-fatal unintentional injuries in older people. The use of Exergames (active, gamified video-based exercises) is a possible innovative, community-based approach. This study aimed to determine the effectiveness of a tailored OTAGO/FaME-based strength and balance Exergame programme for improving balance, maintaining function and reducing falls risk in older people.

**Methods:**

A two-arm cluster randomised controlled trial recruiting adults aged 55 years and older living in 18 assisted living (sheltered housing) facilities (clusters) in the UK. Standard care (physiotherapy advice and leaflet) was compared to a tailored 12-week strength and balance Exergame programme, supported by physiotherapists or trained assistants. Complete case analysis (intention-to-treat) was used to compare the Berg Balance Scale (BBS) at baseline and at 12 weeks. Secondary outcomes included fear of falling, mobility, fall risk, pain, mood, fatigue, cognition, healthcare utilisation and health-related quality of life, and self-reported physical activity and falls.

**Results:**

Eighteen clusters were randomised (9 to each arm) with 56 participants allocated to the intervention and 50 to the control (78% female, mean age 78 years). Fourteen participants withdrew over the 12 weeks (both arms), mainly for ill health. There was an adjusted mean improvement in balance (BBS) of 6.2 (95% CI 2.4 to 10.0) and reduced fear of falling (*p* = 0.007) and pain (*p* = 0.02) in the Exergame group. Mean attendance at sessions was 69% (mean exercising time of 33 min/week). Twenty-four percent of the control group and 20% of the Exergame group fell over the trial period. The change in fall rates significantly favoured the intervention (incident rate ratio 0.31 (95% CI 0.16 to 0.62, *p* = 0.001)). The point estimate of the incremental cost-effectiveness ratio (ICER) was £15,209.80 per quality-adjusted life year (QALY). Using 10,000 bootstrap replications, at the lower bound of the NICE threshold of £20,000 per QALY, there was a 61% probability of Exergames being cost-effective, rising to 73% at the upper bound of £30,000 per QALY.

**Conclusions:**

Exergames, as delivered in this trial, improve balance, pain and fear of falling and are a cost-effective fall prevention strategy in assisted living facilities for people aged 55 years or older.

**Trial registration:**

The trial was registered at ClinicalTrials.gov on 18 Dec 2015 with reference number NCT02634736.

**Electronic supplementary material:**

The online version of this article (10.1186/s12916-019-1278-9) contains supplementary material, which is available to authorized users.

## Background

Fall-related injuries are the largest cause of accidental death in older people across Europe [1] and the second leading cause of accidental death amongst older people globally [2]. Over 30% of community-dwelling people aged 65 and older and 50% of people aged 80 and over fall at least once per year [3, 4]. People living in retirement villages/assisted living facilities fall frequently [5]. Those identified as frail fall more frequently than those who are classified as vigorous [6, 7]. Falls are associated with admission to residential care homes, reduced functioning, psychological problems such as fear of falling and loss of confidence leading to social isolation and increased dependency. The direct and indirect costs of falls are substantial, for example, estimated costs in the UK NHS are in excess of £2.3 billion per year [8].

There is strong evidence that strength and balance-based exercises reduce falls by up to 42% [3, 9, 10] and that strength and balance exercise as a stand-alone intervention may be the most cost-effective approach to fall prevention at a population level [11]. Although there are few studies in assisted living facilities, a cluster randomised controlled trial of group strength and balance exercise in retirement villages in Australia showed a reduction of 22% falls in the intervention group compared to the control [5]. Exercise as a means of fall prevention and for the promotion of independence has been welcomed by older people, as a positive step that individuals can take for themselves [12]. Reviews of community-based fall prevention indicates that strength and balance exercise need to be tailored, progressive and of adequate dose (50 h) [3, 13, 14]. Sherrington et al. reveal that exercise programmes that are of a higher dose (more than 3 h/week) have larger effects [14]. Such training can be costly and inaccessible to older adults [15]. The repetitive nature of these exercises may also discourage older adults to exercise in the home setting, thereby rendering the intervention ineffective [16]. Uptake and adherence to exercise programmes is low, and appropriate levels and progression are often not adequately prescribed [5, 17]. Fear of falling can lead to restriction or avoidance of daily activities, loss of independence, depression and a reduction in quality of life [18]. In frailer older adults and for those living in institutional settings, there is also the risk that unsupervised exercise can increase risk of falls [16, 19]. A number of reviews of exercise to improve function in frailer older adults recommend supervision to ensure progression and effectiveness [20, 21]. Thus, there are compelling reasons to find interventions that are both effective and safe for this population.

Exergaming (active video games which combine gameplay with physical exercise and may also incorporate types of virtual reality simulations) may be a feasible tool for older people to improve exercise uptake, challenge and progression [22]. There is growing evidence that Exergaming may also improve function and adherence and provide other health outcomes [4, 23] and that such technology-based approaches can be attractive to older people [24]. The advantages of using gaming systems to deliver exercise are that they can be immersive, entertaining and enjoyable, potentially improving adherence and frequency/duration of the exercise programme [25, 26]. The gamified elements of Exergames (levels, points, progress) may also encourage uptake and adherence to exercise [27, 28]. The feedback on progress alongside comparison or competition with other players may be persuasive and motivate longer-term use [29, 30]. A recent meta-analysis of effectiveness of virtual reality (VR) games for fall prevention in older people found positive effects on balance and fear of falling compared with no intervention; VR games were also concluded to be superior to conventional treatment [31]. But a high risk of bias, small sample sizes and large variability between methods and interventions mean evidence remains inconclusive and further research is needed. Other systematic reviews of virtual reality exercise programmes, Exergames and technology-based exercise interventions for older people [32–34] present similar conclusions that these interventions have potential, but larger and more rigorous studies are required to make more definitive conclusions regarding the effectiveness of Exergames to improve outcomes such as fall rate and fall risk.

The development and design of the Exergames used in this study were previously tested in a feasibility study [25, 35]. We followed the UK Medical Research Council (MRC) Guidance for Developing and Evaluating Complex Interventions [36] that recommends: identifying the evidence base and relevant theory; modelling process and outcomes; and then testing through feasibility or pilot studies prior to a full-scale evaluation [36].

Exergame programmes were planned to be carried out on a one-to-one basis (i.e. one person playing the Exergames at a time, supervised for research purposes by a physiotherapist or a physiotherapist assistant) either as part of a group setting or individually. Tailoring the Exergame programme and setting realistic, person-centred goals that are continuously reassessed to ensure personalisation and progression were also deemed important [37–39]. Understanding the health benefits of exercise and having self-belief in one’s ability to exercise (self-efficacy) are also necessary [41, 42]. There are also practical considerations for the delivery of the intervention such as ease of access to the exercise, particularly for frailer older adults unwilling or unable to travel [15, 43].

This trial investigated the effectiveness of a suite of Exergames that were developed with, and for, older people to improve function and reduce the risk of falls [25]. A series of games were co-created with older adults, therapists and software designers [25], which are based on OTAGO and FaME exercises for older people (strength and balance exercises with demonstrated effectiveness to reduce falls) [44–46]. The Exergames draw on self-determination theory and gamification to aid uptake, motivation and adherence to the Exergame programme [40]. Evaluation of this intervention is particularly timely given the current emphasis on healthy ageing and prevention [46, 47]. A cluster randomised trial design was adopted as individual randomisation within assisted living facilities would have been open to contamination bias. The research hypothesis was that a 12-week tailored programme of strength and balance Exergames will improve balance in people aged 55 years and older, in assisted living facilities.

### Study objectives

The primary objective of this study was to evaluate the effectiveness of a tailored 12-week fall prevention Exergame programme on balance in adults aged 55 years or older as assessed by the Berg Balance Scale [48].

The secondary objectives were to investigate the effectiveness of the Exergame programme on fear of falling, lower limb function, self-reported physical activity, fall risk, pain, mood, fatigue, cognition, healthcare utilisation, health-related quality of life (HRQoL) and fall rates during a 3-month follow-up.

## Methods

### Design

This was a multi-centre, cluster randomised (1:1 ratio) controlled trial comparing fall prevention Exergames plus standard care (physiotherapist advice and leaflet) against standard care only in adults aged 55 years or older, dwelling in assisted living facilities (a cluster comprised one facility).

### Study setting and participants

Clusters were assisted living facilities (also known as sheltered housing facilities or specialist housing) for people in Greater Manchester or Glasgow, identified via a national housing website [49]. Clusters were eligible if they housed residents aged ≥ 55 years, were willing to take part as agreed by their managers and had sufficient communal space (i.e. > 10 m^2^) to exercise without obstacles. The facilities comprised individual flats and bungalows (ranging from 28 to 80) with residents aged between 45 to 102 years. Each facility had a communal lounge where residents would meet to undertake the intervention. Between January and May 2016, 18 assisted living facilities were invited to participate in geographic areas that included a range of levels of deprivation in urban and semi-urban areas. Eligibility criteria for participants are presented in Table 1.

**Table 1:** Participant eligibility criteria for the study

Inclusion criteria	Exclusion criteria
Aged ≥ 55 years	Acute illness
Mental capacity (assessed by trained healthcare professional) to give informed consent	Severe congestive cardiac failure
Able to speak English sufficiently to understand exercise instructions	Uncontrolled hypertension
Registered with a primary care general practice	Recent fracture or surgery in past 6 months
Able to watch television with or without glasses from 2 m distance	On waiting list to have orthopaedic surgery
Able to use gaming technology safely as assessed by research physiotherapists (i.e. able to stand with support of aids and follow game instructions)	Myocardial infarction or stroke in the past 6 months
	Dependence on wheelchair use
	Severe auditory or visual impairment(s)
	Peripheral neuropathy or other uncontrolled medical conditions likely to compromise the ability to exercise
	Current use of gaming technology to exercise

Managers at the assisted living facilities invited residents to attend an information session about the Exergames and the study, presented by ES or AM. Trained research staff and research physiotherapists initially identified residents who appeared to meet inclusion criteria, and ascertained if they were willing to receive information about the study, before providing them with participant information sheets and consent forms. After at least 24 h, participants were consented, fully assessed for inclusion as per protocol and underwent physical assessment by a research physiotherapist. All participants’ general practitioners (GP) were notified of study participation and eligibility criteria were verified using GP records.

### Cluster randomisation and blinding

To reduce the potential for selection bias, baseline assessments took place before randomisation. Cluster randomisation of the units was based on blocks of two in each location (Manchester; Glasgow), matched according to readiness to deliver the intervention, and the number of consented participants per facility. Recruitment was staggered, with Manchester sites recruiting first. Randomisation was computer-generated (using Stata 14) [50] and performed by the Lancashire Clinical Trials Unit (CTU). Clusters were enrolled and assigned by ES and DS. Blinding of the assessors at the 12-week assessment was not possible for two out of the three assessors due to participants revealing the group allocation of their assisted living facility/cluster. Data analysis was performed by two unblinded researchers at the CTU (CJS, VB) based on an a priori statistical analysis plan. Recruitment commenced in Manchester in January 2016 and ended in Glasgow in June 2016, with the final follow-up being completed in November 2016.

### Description of the interventions

The Control and Exergame group interventions are summarised in Table 2.

**Table 2:** Summary of the control and Exergame group interventions

Description of intervention	Control group	Exergame group
AGE UK Staying Steady Falls Prevention Leaflet [51]	Yes	Yes
OTAGO Strength and Balance Home Exercise Leaflet [52] and advice from physiotherapist to undertake 3 OTAGO-based exercises 3 times per week for 12 weeks	Yes	Yes
Tailored Exergame programme supervised by physiotherapists/physiotherapist assistants 3 times per week for 12 weeks	No	Yes

#### Control

A physiotherapist gave members of the control group standard community fall prevention advice comprising the Age UK Staying Steady leaflet [51] and the OTAGO strength and balance home exercise programme leaflet [52]. Control participants were encouraged to do three preselected (by the physiotherapist) exercises from the OTAGO list over the 12-week period. After 12 weeks, control participants were offered the opportunity to use the Exergame platform to reduce drop out [53].

#### Exergame intervention

In the intervention group, the same standard care as the control group was given. In addition, Exergames were offered three times a week (under the supervision of a physiotherapist or physiotherapist assistant) for 12 consecutive weeks in the assisted living facility communal rooms. Each participant was given a prescribed programme of standardised Exergames that suited the participant’s starting level of ability, with tailored progression (e.g. more Exergames within a session, greater challenge, longer duration) over the 12 weeks.

**Fig. 1:**
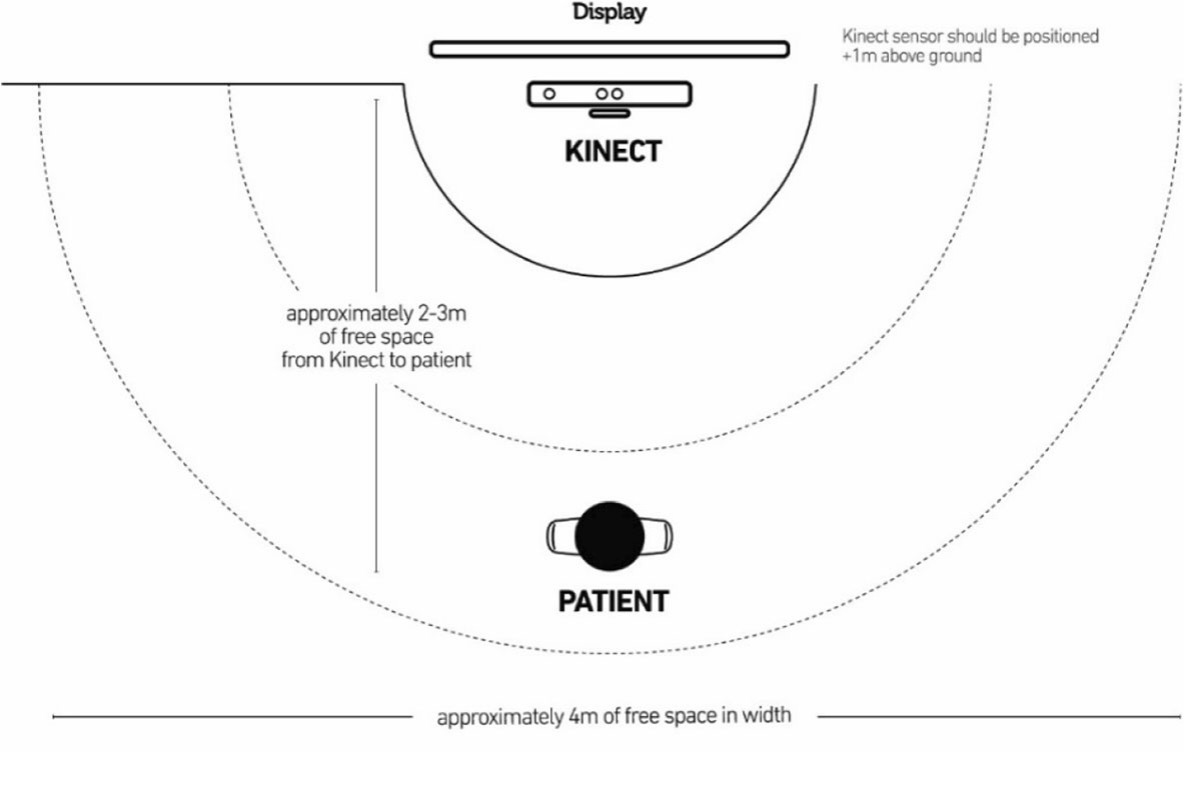
Set-up and positioning of the sensor for Exergame intervention

The physiotherapists and physiotherapist assistants were trained for approximately 30 min in the use of the Exergame system. This consisted of being shown the different games and corresponding exercises and how to set up the laptop and Kinect sensor (version 2; Microsoft Corp., Redmond, WA) and set up a programme for users (Fig. 1). The Kinect sensor requires placement 1 m above the ground and can be manually tilted so that it tracks the participant’s entire body. The participant needs to face the Kinect sensor, with no obstacles in front or to the side to allow free movements, and stand at a distance of 2–3 m.

Physiotherapists and assistants familiarised themselves with the system prior to commencing the trial. Remote technical assistance was provided by MIRA Rehab during the trial. The Exergame system used in this trial was designed to engage and motivate individuals to participate in ‘game’-driven physical activities [54]. In an earlier feasibility study, this Exergame system showed potential to improve balance and increased engagement through motivational design [35]. The Exergame system enables interaction with the user by giving verbal and subtitled feedback on correct movements and any adjustments that may be required. It utilises the Microsoft Kinect (Microsoft Corp., Redmond, WA), a 3D motion tracking device that does not require handheld controls. This tracks the user’s performance and records parameters such as frequency and duration of use. Individual exercise programmes can be tailored using a choice of games for lower or upper limb exercises (see Additional file 1: Tables S1, S2 and Figure S2). The system also supports remote monitoring, whereby the user’s exercise programme (and progress) can be remotely viewed and adjusted by a remote supervisor, but this was not evaluated during this study.

### Outcome measures

Assessments were completed with individual participants at baseline and 12 weeks by trained physiotherapists and included a series of standardised tests and questionnaires (Table 3).

**Table 3:** Outcome measures

Primary outcome	Times at which assessed
Berg Balance Scale (BBS) [55]	Baseline, 12 weeks
Secondary outcomes	
Falls (fall diary) [56]	Daily self-report, posted monthly for 3 months
Adherence: frequency, duration, number of sessions [54]	Recorded at each use of Exergame
Timed Up and Go (TUG) [57]	Baseline, 12 weeks
Fall risk score (FRAT) including VAS pain and VAS fatigue [58]	Baseline, 12 weeks
Physical Activity Scale for the Elderly (PASE) [59]	Baseline, 12 weeks
Addenbrooke’s Cognitive Examination III (ACEIII) [60]	Baseline, 12 weeks
Short Falls Efficacy Scale-International (Short FES-I) [61]	Baseline, 12 weeks
Geriatric Depression Scale (5-item GDS) [62]	Baseline, 12 weeks
Health-related quality of life (HRQoL), Euro-QoL EQ-5D-5L [63, 64]	Baseline, 12 weeks
Monetary costs of health care utilisation following falls [65]	Daily self-report calendar, posted monthly for 3 months and follow-up phone calls [56]
Usability and acceptance of Exergames (system usability scale (SUS) [66] and technology assessment model (TAM)) [67]	12 weeks (Exergame group only)

Balance at 12 weeks post-baseline, measured by the Berg Balance Scale [55], was the primary outcome measure.

A 3-month follow-up of self-reported participant falls (baseline assessment) was conducted using daily fall calendars that were posted monthly to the researchers. Participants who reported a fall during the previous month were contacted by telephone to record details of the fall [56].

The trial protocol is registered on ClinicalTrials.gov reference: NCT02634736.

### Sample size

A sample size calculation (5% two-sided significance level, 90% power) to detect a between-group difference in mean clinically important change in the Berg Balance Scale (BBS) of 8 points [68], indicated a sample size of 23 participants in each group was needed. Assuming a common standard deviation (SD) of 8.0 (conservative estimate from a pilot trial) [35], using an independent-samples *t*-test and assuming a conservative intraclass correlation coefficient (ICC) of 0.05 and a cluster size of 9 participants, this leads to a design effect of 1.4 and sample size of 33 per arm. In our pilot study, 22/24 (92%) provided outcome data at week 6. Conservatively, we assumed a 75% retention rate at week 12. Therefore, at least 5 assisted living facilities in each arm (a total of 10 assisted living facilities), with a site average of 9 participants recruited, were required. However, to allow for unit attrition or limited participation, we recruited a total of 18 assisted living facilities.

### Data collection and analysis

Participant characteristics assessed at baseline included age, sex, ethnicity, employment, marital status, socioeconomic status (Index of multiple deprivation of participants’ assisted living facility postcode), fall history and self-reported vision. Outcome measures (Table 3) were assessed at baseline and 12 weeks.

### Statistical analysis

Our primary analysis was intention-to-treat; therefore, we included all participants willing to provide outcome data irrespective of their engagement with the Exergame or control intervention. We implemented this using a complete case analysis, and no imputation of missing outcome data was performed. However, a sensitivity analysis on the primary outcome variable was performed using multiple imputation (using a linear regression model, with age, BBS at baseline, location and group as predictors) [69] if there was more than 10% missing data or a between-group difference of more than 10% in the percentages with missing data. To account for the cluster randomisation, linear mixed effects modelling for the primary and secondary outcomes was used to compare the two groups. The group indicator was included in each outcome model as the focus of the analysis and adjustment was performed for the following variables: assisted living facility unit (random effect), baseline measures of the corresponding outcome variable (fixed effect), location indicator (Manchester; Glasgow; fixed effect). To investigate potential differences in effectiveness between locations, we added a location-by-intervention interaction term to the model for the primary outcome measure.

Fall data were summarised as recommended by ProFaNE [70] using the number of falls, number of non-fallers/single fallers/multiple fallers and fall rate per person-year. We investigated the effect of the Exergame intervention on fall rate by estimating the incidence rate ratio (IRR) using Mantel-Haenszel methods, stratified by match pair [71].

A significance level of 5% was used, and effectiveness estimation included both point and 95% confidence interval estimates. All analyses were performed using Stata 14 [50].

### Economic analysis

The primary objective of the economic analysis was to assess the incremental cost-effectiveness of Exergames compared to treatment as usual (TAU). In this case, TAU was a visit from a physiotherapist to explain the Otago Exercise Programme (OEP) and to talk through a leaflet on fall prevention and an additional leaflet explaining the OEP recommended exercises. The control arm was asked to undertake their individually recommended exercises three times a week in their leisure time. However, TAU was not the full OEP (which may be the case in a non-trial setting) that is delivered by trained personnel at home [72] or in group setting [73]. The analysis was conducted alongside the Exergame trial from the perspective of the English National Health Service (NHS).

Quality-adjusted life years (QALYs) were calculated based on EQ-5D-5L using the area under the curve method assuming linear extrapolation of utility between time points. Following Hunter et al., we calculated the QALYs at the individual level (rather than at the group level) [74]. Health care services resource-use data were collected during the study and combined with relevant unit cost data for the financial year 2015–2016 to calculate the total health service costs incurred over the study period.

Multiple-imputation techniques were used for the main analysis presented here. Following White et al. and Faria et al., we used 15 imputations (as the missing data percentage was approximately 15%) [75, 76].

The incremental cost-effectiveness ratio (ICER) was calculated, adjusting for baseline EQ-5D-5L index score following Manca et al. [77]. We further adjusted for gender and age at baseline as well as identifiers for each of the residential homes.

## Results

### Study participants

**Fig. 2:**
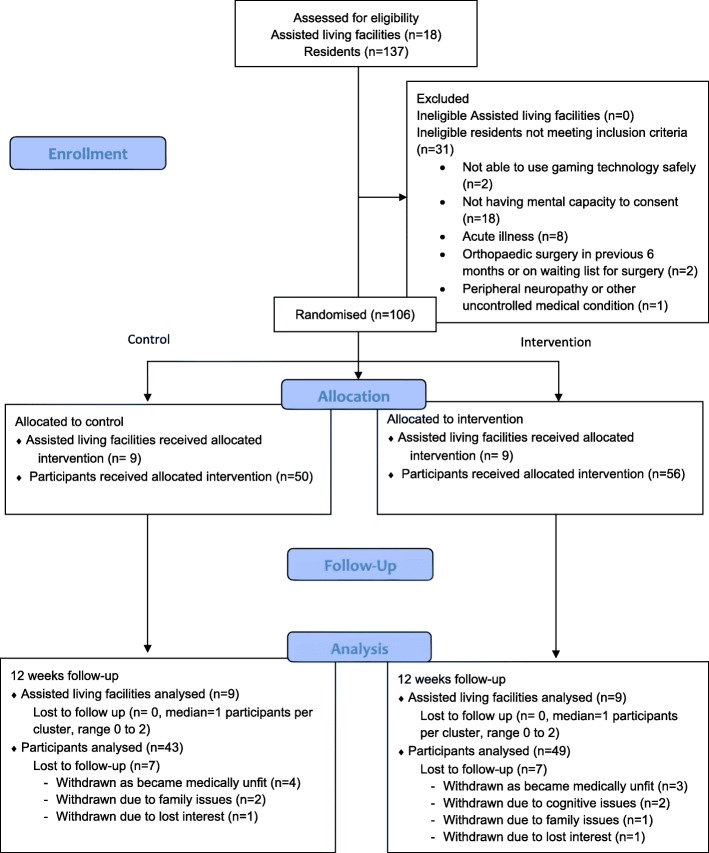
CONSORT flow diagram for cluster RCT

Figure 2 shows the flow of participants through the study. Eighteen assisted living facilities gave permission at a managerial level to take part in the study and were randomised to control (*n* = 9) or to Exergame intervention (*n* = 9). The assisted living facilities ranged from smaller facilities with 19 occupied flats to larger facilities with 80 occupied flats. Following invitation by the managers of the facilities, 137 adults aged 55 years or older expressed an interest in participating in the study. Of these, 31 did not meet the inclusion criteria mostly related to participants not having the mental capacity to consent (Fig. 2). All assisted living facilities randomised were retained in the trial.

The 106 participants’ baseline characteristics are presented in Table 4. Almost 80% of the participants were female. Mean ages of control and Exergame groups were similar (77.8 (SD = 10.2) vs 77.9 (SD = 8.9) years respectively), and there was a good balance between the groups in terms of ethnicity, employment and marital status. More participants from the Exergame group reported a fall in the previous year (58%) compared to control (42.9%) and Glasgow had higher index of multiple deprivation scores than Manchester sites, particularly in the Glasgow Exergame group.

**Table 4:** Baseline characteristics

Assistive living facility (ALF) characteristics	Control ‘usual care’ *n* = 9	Intervention ‘Exergames’ *n* = 9
Location, *n*		
Manchester	6	6
Glasgow	3	3
Manchester IMD rank^a^, median (range, IQR)	14,844 (3319–23,972, (8839.0–18,169.25))	19,931 (4647–26,933) (11,715.8–26,543.0)
Glasgow SIMD rank^b^, median (range, IQR)	2788 (1382–5907) (2085.0–4347.5)	1004 (149–5100) (576.0–3052.0)
Manchester IMD decile, median (range, IQR)	5 (2–9)	6.5 (2–9)
Glasgow SIMD decile, median (range, IQR)	4 (2–4)	2 (1–8)
Participant characteristics	Control ‘usual care’ *n* = 50	Intervention ‘Exergames’ n = 56
Participants, *n*	50	56
Manchester	32	41
Glasgow	18	15
Female sex, *n* (%)	38 (76.0)	45 (80.4)
Age, years (SD/range)	77.8 (10.2/58–101)	77.9 (8.9/58–96)
Ethnicity, *n* (%)		
White British or Irish	50 (100)	52 (92.9)
Asian or Asian British	0 (0.0)	1 (1.8)
Mixed	0 (0.0)	2 (3.6)
Other ethnic groups	0 (0.0)	1 (1.8)
Employment, *n* (%)		
Retired	47 (94.0)	54 (96.4)
Doing voluntary work	1 (2.0)	1 (1.8)
Unemployed through sickness/disability	2 (4.0)	1 (1.8)
Marital status, *n* (%)		
Single, never married	7 (14.0)	10 (17.9)
Married/living with partner	8 (16.0)	2 (3.6)
Divorced	6 (12.0)	10 (17.9)
Separated	1 (2.0)	1 (1.8)
Widowed	28 (56.0)	33 (58.9)
1 year history of falls, *n* (%)		
No falls reported (non-fallers)	21 (42)	32 (57)
1 fall reported (single fallers)	14 (28)	7 (13)
> 1 fall reported (multiple fallers)	15 (30)	17 (30)
Self-reported vision, *n* (%)		
Excellent	4 (8)	7 (13)
Good	24 (48)	22 (39)
Fair	16 (32)	19 (34)
Poor	5 (10)	7 (13)
Very poor	1 (2)	0 (0)
Registered blind	0 (0)	1 (2)

IMD and SIMD decile score ranges 1–10, where 1 is the least deprived and 10 the most deprived

^a^The IMD (2015) rank is the Index of Multiple Deprivation and combines a number of indicators, chosen to cover a range of social, economic and housing issues, into a single deprivation ranked score, the lower the score the more deprived

^b^The SIMD (2016) rank is the Scottish equivalent of the IMD rank

### Study retention and adherence

Retention levels at 12 weeks was 86.8%. The most reported reasons for withdrawing from the study were ‘being medically (physically/mentally) unfit’ (*n* = 9), ‘family issues’ (*n* = 3) and ‘loss of interest’ (*n* = 2) (Fig. 2).

The Exergame participants attended a mean number of 25 (SD = 8.5) of a total of 36 sessions offered over the 12-week study period (a mean of 2 out of the 3 weekly offered sessions). Attendance at 12 weeks was 87.5% for the intervention group. The mean Exergame total exercise time at the end of the 12 weeks was 359 min (SD 151.2).

### Effect of intervention

#### Primary outcome

Using ITT analysis, over 12 weeks, the Exergame intervention had a significant positive impact on balance as measured by BBS, relative to control [6.2 (95% CI 2.4 to 10.0; *p* = 0.003)]. The mean positive change of BBS from baseline was 2.9 points (SD 8.5) for the Exergame group, representing a 4.3% improvement from baseline, whilst the control group deteriorated by a mean of 2.8 (SD 6.5) points.

The estimated intracluster correlation coefficient for the BBS at 12 weeks was 0.08. There was no evidence that the effect of Exergames differed between the two locations (*p* = 0.39). As there was more than 10% missing outcome data, a sensitivity analysis was performed using multiple imputation of the missing values of BBS at week 12. Findings were not sensitive to ignoring the missing data and performing a complete case analysis, with the estimated impact of Exergames on BBS at week 12 being almost identical when using multiple imputation (6.2, 95% CI 2.7 to 9.8; *p* = 0.002), thus supporting the ITT results.

#### Secondary outcomes

Relative to controls, at 12 weeks the Exergames had a positive impact on fear of falling measured by Short FES-I (adjusted mean difference = − 2.7, 95% CI − 4.5 to − 0.8, *p* = 0.007) and VAS pain scale (− 12.1, 95% CI − 22.3 to − 1.8, *p* = 0.024). No statistically significant impact of the Exergame intervention was found on any other secondary outcomes (Table 5).

**Table 5:** Primary and secondary outcome measures at baseline and 12 weeks

	Baseline (*N* = 106)	12 weeks (*N* = 91)	Adjusted difference*	95% CI	*p*	ICC
BBS (0–56) [SD]			6.18	2.38,9.97	0.003	0.08
Control	40.6 [13.1])	37.6 [14.9]				
Exergames	42.0 [13.5])	44.6 [10.7]				
ACE III (0–100) [SD]			− 0.83	− 4.10,2.45	0.60	< 0.001
Control	73.5 [12.1]	80.2 [12.9				
Exergames	77.4 [15.6]	81.4 (14.2)				
7-item FES-I (7–28) [SD]			− 2.69	− 4.52, − 0.85	0.007	0.12
Control	11.6 [4.5]	12.8 [4.8]				
Exergames	11.0 [4.2]	9.8 [3.4]				
VAS pain scale (0–100) [%]			− 12.07	− 22.31, − 1.83	0.024	< 0.001
Control	24.3 [26.5]	34.4 [30.5]				
Exergames	23.9 (25.9)	21.9 [27.7]				
VAS fatigue scale (%)			− 6.63	− 20.58, 7.32	0.33	0.10
Control	32.4 [26.6]	39.2 [28.0]				
Exergames	37.5 [31.2]	34.6 [31.3]				
FRAT (0–5) [SD]			− 0.15	− 0.55, 0.26	0.46	0.05
Control	2.4 [1.3]	2.4 [1.4]				
Exergames	2.3 [1.1]	2.2 [1.2]				
5-item GDS (0–5) [SD]			0.21	− 0.24, 0.65	0.34	0.04
Control	1.4 [1.3]	0.98 [1.1]				
Exergames	1.2 [1.3]	1.0 [1.3]				
TUG (s) [SD]			− 0.82	− 3.62 to 1.98	0.54	< 0.001
Control	20.4 [14.3]	20.7 [13.3]				
Exergames	17.5 [9.0]	17.8 [10.4]				
EQ5D5L-VAS (0–100) [SD]			3.86	− 6.46 to 14.17	0.44	0.05
Control	71.2 [18.3]	67.2 [22.7]				
Exergames	71.2 [21.4]	70.6 [21.1]				
PASE score (0–400) [SD]			− 0.97	− 19.54 to 17.60	0.91	0.10
Control	66.8 [38.2]	72.3 [41.5]				
Exergames	75.1 [50.5]	77.5 [43.1]				

Unless otherwise stated, means (SD) are reported

*BBS* Berg Balance Scale, *SD* standard deviation, *ACE III* Addenbrooke’s Cognitive Examination–III, *7-Item FES-I* 7-Item Falls Efficacy Scale-International, *VAS* visual analogue scale, *FRAT* Falls Risk Assessment Tool, *GDS* Geriatric Depression Scale, *TUG* Timed Up and Go, *EQ5D5L-VAS* European Quality of Life 5 Dimensions -Visual Analogue Scale, *PASE* Physical Activity Scale for the Elderly

*Outcome (e.g. BBS) at 12 weeks adjusted for baseline value of outcome measure, region (Manchester/Glasgow) and site (random effect)

### Follow-up fall incidence

A total of 55 falls were self-reported by the 106 participants during the 3-month follow-up. Of these, 38 falls were reported by the control group (12 fallers (24%) of whom 5 were single fallers and 7 were multiple fallers), an incident rate of 3.11 falls per person-year. Seventeen falls were self-reported by the Exergame group (11 fallers (20%) of whom 8 were single fallers and 3 multiple fallers), an incident rate of 1.26 falls per person-year. The incident rate ratio (IRR) of falls between groups was 0.31 (95% CI 0.16 to 0.62, *p* = 0.001) in favour of Exergames.

### Health economic outcomes

Exergames were associated with a mean incremental total cost increase of £101.84 (95% CI − £7.42 to £211.11) and a mean incremental QALY gain of 0.007 (95% CI − 0.003 to 0.016) (Table 6). Whilst there were no statistically significant differences in costs or QALYs between the control and treatment arms during bootstrapping, the point estimate of the ICER was £15,209.80 per QALY.

**Table 6:** Cost-effectiveness analysis comparing Exergames with OEP leaflet control

	Coeff.	Bootstrapped standard error	Bootstrapped 95% confidence interval
Incremental cost (£)	101.84	55.75	[− 7.42 to 211.11]
Incremental QALYs	0.007	0.004	[− 0.003 to 0.016]
ICER (£)	15,209.80		

Bootstrapping based on 10,000 replications. Note the ICER is not exactly equal to the ratio due to rounding

**Fig. 3:**
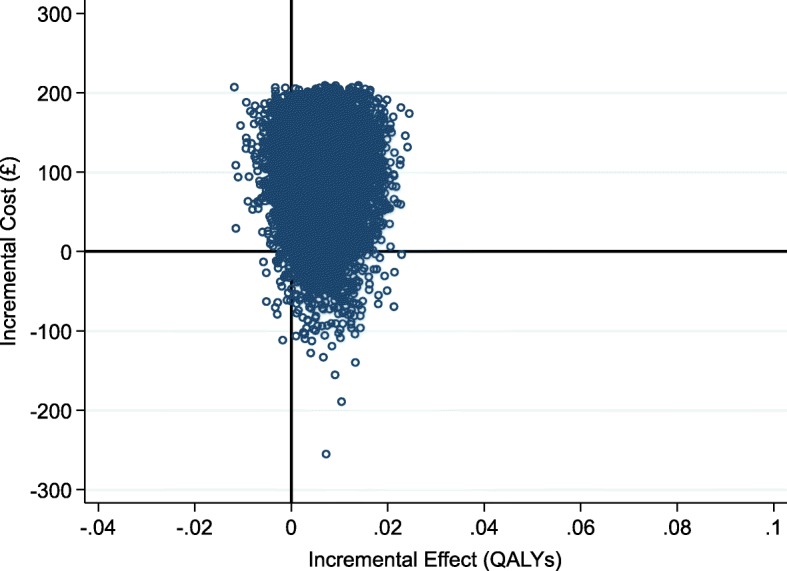
Cost-effectiveness plane for Exergames vs OEP leaflet control

The cost-effectiveness plane (Fig. 3) plots the 10,000 bootstrap replications of incremental cost and QALY estimates, to help illustrate the uncertainty surrounding the point estimates in probabilistic terms. The replications were clustered predominantly in the north-east quadrant, reflecting the point estimates that Exergames resulted in a positive health gain, but at an increased cost. Exergames resulted in an incremental QALY gain in 91.5% (9151 out of 10,000) of bootstrap replications, and a higher cost than controls in 95% (9490 out of 10,000) of replications.

**Fig. 4:**
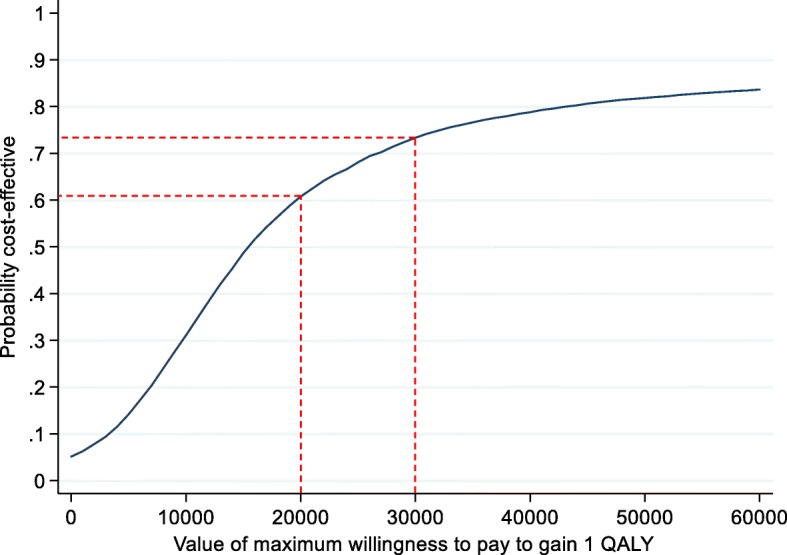
Cost-effectiveness acceptability curve of willingness to pay for one additional quality-adjusted life year (£)

The cost-effectiveness acceptability curve (CEAC; Fig. 4) demonstrates how the probability that Exergames are cost-effective increases with the decision-maker’s willingness to pay. At the lower bound of the NICE threshold [78] of £20,000 per QALY, there was a 61% probability of Exergames being cost-effective. This rose to 73% at the upper bound of £30,000. Compared with controls, Exergames were likely to be cost-effective in 50% or more cases if decision makers are willing to pay approximately £15,500 for one additional QALY.

### Exergame usability and acceptance

Participants scored the Exergame system (set up by the physiotherapist or assistant) on the technology assessment model (1 = strongly disagree to 7 = strongly agree) as easy to use (mean = 6.3, SD = 1.4) and useful (mean = 5.9, SD = 1.9). Participants had a favourable attitude (mean = 6.6, SD = 1.2) and indicated they intended to use the Exergame system in the future if it were to become available (mean = 5.7, SD = 2.2).

The mean system usability scale (range 0–100) at 12-week assessment was 82.4 (SD = 15.5) indicating high usability [79].

No major protocol deviations or unexpected adverse events occurred during the study period.

## Discussion

This is the first cluster RCT of Exergames in community-based, assisted living facilities. Provision of Exergames for strength and balance exercises over 12 weeks to residents aged 55 years and older of these facilities significantly improved balance (as measured by the BBS), pain (VAS) and fear of falling (Short FES-I). Furthermore, an IRR = 0.31 for falls over a 3-month follow-up indicates an unexpected significant effect for this fall reduction.

The trial was powered on the minimum detectable change (MDC) of 8 points on the BBS from a trial of dependent older people (living in residential care homes) of somewhat older age than those in this trial [68]. However, other studies have found that the MDC for more physically independent older people receiving physiotherapy was rather less (3.2 points [80] and 4.9 points [81]). Furthermore, it has recently been advised that studies should not be powered on distribution-based statistics, such as the MDC [82], but should primarily use anchor-based methods and/or expert opinion to estimate the minimally important difference (MID).

There has been no research to determine the MID for the BBS on a population similar to ours, but Saso et al. [81] found an MID of 6 points for people early after stroke and Godi et al. [83] found an MID of 7 points for a groups of older people with balance deficits undergoing rehabilitation. Given the nature of the population in our trial, the MID would be expected to be not greater than 6 and perhaps a little smaller. The estimated difference in change between the groups for balance (BBS 6.2 points) is therefore plausible as an important difference, if only slightly exceeding the likely MID. However, the 95% confidence interval for this effect is relatively wide (2.4 to 10.0) so further research is recommended to provide further evidence as to the magnitude of the effect.

The results of our economic evaluation imply that Exergames are likely to be cost-effective compared to control (physio advice and the Otago Exercise Programme leaflet). These findings were robust, controlling for baseline characteristics using multiple imputation or complete case analysis, and choice of methodology to derive utility values from the EQ-5D-5L instrument. In the primary analysis, the ICER was £15,209.80 per QALY, and there was a 61–73% probability of Exergames being cost-effective at NICE thresholds [78] of £20,000–£30,000 per QALY. It should be noted that the full Otago Exercise Programme consists of a set of three times per week progressive exercises, over a 12-month period and a walking plan with multiple home visits by an instructor [45]. Therefore, traditional Otago Exercise Programme costs would be much higher than those proposed in this current 12-week study due to the additional labour costs, on-going training, travel, telephone calls and overhead costs.

This study demonstrated consistent findings between the locations indicating the potential transferability of the Exergame system. Retention of the Exergame participants was high (87%) compared to other exercise programmes for older people [84], with a mean attendance at Exergame sessions of around two thirds (69%). Although there is no current consensus on the cut-off points for levels of high, moderate or low adherence [85], this may be considered a beneficial level of attendance that resulted in improvements in balance, the primary outcome measure and also falls. Participants also reportedly liked the Exergames and found them easy to use (albeit, after it had been set up by the therapists, so this may refer to their understanding and playing of the Exergames). These are important factors when considering not just the effectiveness of the intervention but also whether people will actually want to use it in the long term [86]. We recorded no adverse events related to the 12-week intervention.

### Strengths and limitations

Baseline assessment was undertaken pre-randomisation, thus limiting the potential for selection bias. Sample sizes were achieved and high retention rates suggest little effect of attrition bias. Recruitment across multiple locations provides some evidence of generalisability across assisted living facilities and should reduce risks of performance bias. The Exergames were tested with people aged 55 years or older using rigorous methods in a ‘real-world’ context in communal areas in assisted living facilities rather than in a controlled laboratory-type setting, and the physiotherapists and assistants had no prior experience of this type of technology. Benefits of the intervention were that the Exergames were co-designed with older people and therapists and are based on evidence-based exercises (FaME and OTAGO) and gamification health psychology [27] and can be tailored to a participant’s level of ability, preference and progression (i.e. different upper and lower limb exercises could be matched to different games and changed throughout the intervention period to maintain interest).

Cluster randomisation was essential to ensure that intervention contamination did not occur, but this also made blinding of the assessors difficult. Therefore, despite the use of objective measures and standardised participant instructions, it is possible that some of the improvements at follow up may have resulted from detection bias and so the results should be interpreted with caution. Blinding of the data analysts would also have been difficult, although there is sparse evidence that this may have affected outcomes [87]. An a priori analysis plan was agreed and executed to minimise risk of reporting bias.

The incidence rate ratio for falls over the 3-month follow up (IRR = 0.31) significantly favours the Exergame group and this is in line with community-based RCTs testing the exercises that the Exergames were based on [3, 9, 52]. But falls were only followed up for 3 months, and falls were self-reported by the participants. Recall bias could be present, and it is recommended in future studies that falls are prospectively followed up for 1 year as per the ProFaNE consensus guidelines [70]. A further limitation is that the majority of the participants were women and of white, British ethnic origin. Future studies need to target ethnic groups and more men. Changes in physiotherapy staffing (one change in Manchester, two changes in Glasgow) could have also affected the delivery of the intervention, as it was observed that it took around 2 weeks for the therapists to become confident in setting up and delivering the Exergames to the intervention group. There tended to be more remote technology support required during these early weeks to ensure the sessions ran smoothly. These support issues caused short delays in the delivery of the Exergames which could have reduced both the duration of the session and the enjoyment level of the participants.

Attendance at the Exergame sessions was generally good, but the amount of time spent exercising (mean 33 min per week) was well below the recommended level of 150 min per week of moderate aerobic activity for adults and older people [46]. This appears to be an issue, even in well-designed community exercise RCTs [88]. However, in one recent cluster RCT, increased levels of physical activity were self-reported, albeit below the recommended levels, and the fall rate was reduced [89].

Future studies are needed to investigate the optimum intensity, progression and long-term adherence to Exergame programmes. Moreover, such studies should investigate improvements in balance using clinical measures (e.g. BBS) with also include an instrumental measurement of static and dynamic balance (e.g. posturography) and include other outcomes such as lower limb muscle strength [14], fall rates, fall risk, fatigue and habitual physical activity. In particular, a definitive trial is recommended to evaluate the cost-effectiveness of the implementation of the Exergames with a primary outcome of fall rates, and costs per QALY along with a process evaluation comparing traditional delivery of exercise [86]. More research is also required to test the effectiveness of Exergames in other settings such as care homes, within early hospital discharge schemes, or to top-up or continue longer-term rehabilitation at home under remote monitoring by clinicians. This is particularly important as, in frailer older adults, unsupervised home exercise may be risky [19].

## Conclusions

The use of technology to reduce falls and to support exercise uptake and adherence in younger and older adults is increasing. This study has demonstrated that an evidence-based programme of Exergames is an acceptable, effective and potentially cost-effective way to improve balance, pain and fear of falling. Exergames may be a scalable intervention to reduce falls (at a personal and societal level).

## Additional file

Additional file 1: **Table S1.** Exercise description and corresponding games. **Figure S1.** An example of a participant Exergame schedule. **Table S2.** Games and description of play. (DOCX 876 kb)

## References

[1] Εuropean Commission (2017). Accidents and injuries statistics.

[2] World Health Organization (2016). WHO fact sheet on falls.

[3] Gillespie LD, Robertson MC, Gillespie WJ, Sherrington C, Gates S, Clemson LM, Lamb SE (2012). Interventions for preventing falls in older people living in the community. Cochrane Database Syst Rev.

[4] van Diest M, Lamoth CJC, Stegenga J, Verkerke GJ, Postema K (2013). Exergaming for balance training of elderly: state of the art and future developments. J Neuroeng Rehabil.

[5] Lord SR, Castell S, Corcoran J, Dayhew J, Matters B, Shan A, Williams P (2003). The effect of group exercise on physical functioning and falls in frail older people living in retirement villages: a randomized, controlled trial. J Am Geriatr Soc.

[6] Todd C, Becker C, Woo J. Falls. In: Michel J-P, Beattie L, Martin F, Walston J, editors. Oxford textbook of geriatric medicine. 3rd ed: Oxford University Press; 2016. DOI: 10.1093/med/9780198701590.001.0001.

[7] Speechley M, Tinetti M (1991). Falls and injuries in frail and vigorous community elderly persons. J Am Geriatr Soc.

[8] National Institute for Health and Care Excellence (2013). Falls: assessment and prevention of falls in older people.

[9] Robertson MC, Devlin N, Gardner MM, Campbell AJ (2001). Effectiveness and economic evaluation of a nurse delivered home exercise programme to prevent falls. 1: Randomised controlled trial. BMJ.

[10] Robertson MC, Campbell AJ, Gardner MM, Devlin N (2002). Preventing injuries in older people by preventing falls: a meta-analysis of individual-level data. J Am Geriatr Soc.

[11] Davis JC, Robertson MC, Ashe MC (2010). Does a home-based strength and balance programme in people aged ≥80 years provide the best value for money to prevent falls? A systematic review of economic evaluations of falls prevention interventions. Br J Sports Med.

[12] Yardley L, Donovan-Hall M, Francis K, Todd C (2006). Older people’s views of advice about falls prevention: a qualitative study. Health Educ Res.

[13] Sherrington C (2008). Effective exercise for the prevention of falls: a systematic review and meta-analysis. J Am Geriatr Soc.

[14] Sherrington C, Michaleff ZA, Fairhall N, Paul SS, Tiedemann A, Whitney J, Cumming RG, Herbert RD, Close JCT, Lord SR. Exercise to prevent falls in older adults: an updated systematic review and meta-analysis. Br J Sports Med. 2016. 10.1136/bjsports-2016-096547. 10.1136/bjsports-2016-096547 27707740

[15] Franco MR, Tong A, Howard K, Sherrington C, Ferreira PH, Pinto RF, Ferreira ML (2015). Older people's perspectives on participation in physical activity: a systematic review and thematic synthesis of qualitative literature. Br J Sports Med.

[16] Nyman SR, Victor CR (2012). Older people’s participation in and engagement with falls prevention interventions in community settings: an augment to the Cochrane systematic review. Age Ageing.

[17] Lord SR, Sherrington C, Cameron ID, Close JCT (2011). Implementing falls prevention research into policy and practice in Australia: past, present and future. J Saf Res.

[18] Delbaere K, Close JCT, Brodaty H, Sachdev P, Lord SR (2010). Determinants of disparities between perceived and physiological risk of falling among elderly people: cohort study. BMJ.

[19] Sherrington C, Lord SR, Vogler CM, Close JC, Howard K, Dean CM, Heller GZ, Clemson L, O'Rourke SD, Ramsay E, Barraclough E, Herbert RD, Cumming RG (2014). A post-hospital home exercise program improved mobility but increased falls in older people: a randomised controlled trial. PLoS One.

[20] Beaudart C, Dawson A, Shaw SC, Harvey NC, Kanis JA, Binkley N, Reginster JY, Chapurlat R, Chan DC, Bruyère O, Rizzoli R, Cooper C, Dennison EM, IOF-ESCEO Sarcopenia Working Group (2017). Nutrition and physical activity in the prevention and treatment of sarcopenia: systematic review. Osteoporos Int.

[21] de Labra C, Guimaraes-Pinheiro C, Maseda A, Lorenzo T, Millán-Calenti JC (2015). Effects of physical exercise interventions in frail older adults: a systematic review of randomized controlled trials. BMC Geriatr.

[22] Vieira ER, Palmer RC, Chaves PHM (2016). Prevention of falls in older people living in the community. BMJ.

[23] Corbetta D, Imeri F, Gatti R (2015). Rehabilitation that incorporates virtual reality is more effective than standard rehabilitation for improving walking speed, balance and mobility after stroke: a systematic review. J Physiother.

[24] Helbostad JL, Vereijken B, Becker C, Todd C, Taraldsen K, Pijnappels M, Aminian K, Mellone S. Mobile health applications to promote active and healthy ageing. Sensors. 2017;17(622). 10.3390/s17030622. 10.3390/s17030622 PMC5375908 28335475

[25] Meekes W, Stanmore EK (2017). Motivational determinants of Exergame participation for older people in assisted living facilities: mixed-methods study. J Med Internet Res.

[26] Lange B, Flynn SM, Rizzo AA (2009). Game-based telerehabilitation. Eur J Phys Rehabil Med.

[27] Dewick P, Stanmore E, Cudd P, de Witte L (2017). Applying Game Thinking to Slips, Trips and Falls Prevention. Harnessing the power of technology to improve lives.

[28] Stanmore E, Mavroeidi A, Meekes W, Skelton D, Sutton C, Benedetto V, de Jong LD, Todd C. Exergames to reduce falls risk in older people in UK assisted living facilities: A multi-centre cluster RCT. Innovation in Aging. 2018;2(Suppl 1):362–63. 10.1093/geroni/igy023.1340. 10.1186/s12916-019-1278-9 PMC6394073 30813926

[29] Uzor S, Baillie L, Skelton DA, Fairlie F, Campos P, Graham N, Jorge J, Nunes N, Palanque P, Winckler M (2011). Identifying barriers to effective user interaction with rehabilitation tools in the home. Human-computer interaction – INTERACT 2011.

[30] Johnson D, Deterding S, Kuhn KA, Staneva A, Stoyanov S, Hides L (2016). Gamification for health and wellbeing: a systematic review of the literature. Internet Interv.

[31] Neri SGR, Cardoso JR, Cruz L, Lima RM, de Oliveira RJ, Iversen MD, Carregaro RL (2017). Do virtual reality games improve mobility skills and balance measurements in community-dwelling older adults? Systematic review and meta-analysis. Clin Rehabil.

[32] Valenzuela T, Okubo Y, Woodbury A, Lord SR, Delbaere K (2018). Adherence to technology-based exercise programs in older adults: a systematic review. J Geriatr Phys Ther.

[33] Skjæret N, Nawaz A, Morat T, Schoene D, Helbostad JL, Vereijken B (2016). Exercise and rehabilitation delivered through exergames in older adults: an integrative review of technologies, safety and efficacy. Int J Med Inform.

[34] Zeng N, Pope Z, Lee JE, Gao Z (2017). A systematic review of active video games on rehabilitative outcomes among older patients. J Sport Health Sci.

[35] Stanmore EK, Skelton DA, Todd C (2015). Acceptability and usability of Exergames designed to improve function in older people. The Gerontologist.

[36] Craig P, Dieppe P, Macintyre S, Michie S, Nazareth I, Petticrew M (2008). Developing and evaluating complex interventions: the new Medical Research Council guidance. BMJ.

[37] Castro CM, King AC, Brassington GS (2001). Telephone versus mail interventions for maintenance of physical activity in older adults. Health Psychol.

[38] French DP, Olander EK, Chisholm A, McSharry J (2014). Which behavior change techniques are most effective at increasing older adults’ self-efficacy and physical activity behavior? A systematic review. Ann Behav Med.

[39] Williams SL, French DP (2011). What are the most effective physical activity self-efficacy and physical activity behaviour intervention techniques for changing- and are they the same?. Health Educ Res.

[40] Deci EL, Ryan RM (2005). Intrinsic motivation inventory (IMI).

[41] McGowan LJ, Devereux-Fitzgerald A, Powell R, French DP. How acceptable do older adults find the concept of being physically active? A systematic review and meta-synthesis. Int Rev Sport Exerc Psychol. 2017;11:1–24.

[42] Hui SSC, Morrow JR (2001). Level of participation and knowledge of physical activity in Hong Kong Chinese adults and their association with age. J Aging Phys Activ.

[43] Stevens JA, Burns ER (2015). A CDC compendium of effective fall interventions: what works for community-dwelling older adults.

[44] Skelton D, Dinan S, Campbell M, Rutherford O (2005). Tailored group exercise (falls management exercise - FaME) reduces falls in community-dwelling older frequent fallers (an RCT). Age Ageing.

[45] Campbell AJ, Robertson MC, Gardner MM, Norton RN, Tilyard MW, Buchner DM (1997). Randomised controlled trial of a general practice programme of home based exercise to prevent falls in elderly women. BMJ.

[46] Public Health England (2017). Falls and fractures: consensus statement and resources pack.

[47] European Innovation Partnership on Active and Healthy Ageing (2016) Action group A2 Renovated Action Plan 2016–2018. Available at https://ec.europa.eu/eip/ageing/sites/eipaha/files/library/renovated_action_plan_2016-2018_ag_a2.pdf. Accessed 17 July 2018].

[48] ClinicalTrials.gov (2018) Bethesda (MD): National Library of Medicine (US). Identifier NCT02634736; Cluster RCT of Falls Prevention Exergames for Older Adults Available at https://clinicaltrials.gov/ct2/show/NCT02634736. Accessed 17 July 2018.

[49] Housingcare.org (2018) Care homes, retirement property and elderly home care UK [online] Available at http://www.housingcare.org/index.aspx. Accessed 17 July 18.

[50] StataCorp (2017). Stata Statistical Software: release 14.

[51] AGE UK (2017). Staying steady: Keep active and reduce your risk of falling.

[52] Campbell AJ, Robertson C (2003). Otago Exercise Programme to prevent falls in older adults.

[53] Steins Bisschop CN, Courneya KS, Velthuis MJ, Monninkhof EM, Jones LW, Friedenreich C, van de Wall E, Peters PHM, May AM (2015). Control group design, contamination and drop-out in exercise oncology trials: a systematic review. PLoS One.

[54] MIRA Rehab (2016). Play your way to recovery.

[55] Berg KO, Maki BE, Williams JI, Holliday PJ, Wood-Dauphinee SL (1992). Clinical and laboratory measures of postural balance in an elderly population. Arch Phys Med Rehabil.

[56] Schwenk M, Lauenroth A, Stock C, Moreno RR, Oster P, McHugh G, Todd C, Hauer K (2012). Definitions and methods of measuring and reporting on injurious falls in randomized controlled fall prevention trials: a systematic review. BMC Med Res Methodol.

[57] Podsiadlo D, Richardson S (1991). The timed “Up & Go”: a test of basic functional mobility for frail elderly persons. J Am Geriatr Soc.

[58] Nandy S, Parsons S, Cryer C, Underwood M, Rashbrook E, Carter Y, Eldridge S, Close J, Skelton D, Taylor S, Feder G, on behalf of the falls prevention pilot steering group (2004). Development and preliminary examination of the predictive validity of the falls risk assessment tool (FRAT) for use in primary care. J Pub Health.

[59] Washburn RA, McAuley E, Katula J, Mihalko SL, Boileau RA (1999). The physical activity scale for the elderly (PASE): evidence for validity. J Clin Epidemiol.

[60] Hsieh S, Schubert S, Hoon C, Mioshi E, Hodges JR (2013). (2013) validation of the Addenbrooke’s Cognitive Examination-III in frontotemporal dementia and Alzheimer’s disease. Dement Geriatr Cogn Disord.

[61] Kempen GI, Yardley L, van Haastregt JC, Zijlstra GA, Beyer N, Hauer K, Todd C (2008). The Short FES-I: a shortened version of the falls efficacy scale-international to assess fear of falling. Age Ageing.

[62] Hoyl MT, Alessi CA, Harker JO, Josephson KR, Pietruszka FM, Koelfgen M, Mervis JR, Fitten LJ, Rubenstein LZ (1999). Development and testing of a five-item version of the geriatric depression scale. J Am Geriatr Soc.

[63] Herdman M, Gudex C, Lloyd A, Janssen M, Kind P, Parkin D, Bonsel G, Badia X (2011). Development and preliminary testing of the new five-level version of EQ-5D (EQ-5D-5L). Qual Life Res.

[64] van Hout B, Janssen MF, Feng Y-S, Kohlmann T, Busschbach J, Golicki D (2012). Interim scoring for the EQ-5D-5L: mapping the EQ-5D-5L to EQ-5D-3L value sets. Value Health.

[65] PSSRU | Unit Costs of Health and Social Care (2016) [Internet]. [cited 2017 Mar 8]. Available from: http://www.pssru.ac.uk/project-pages/unit-costs/2016/

[66] Brooke J, Jordan PW, Thomas B, Weerdmeester BA, McClelland IL (1996). SUS: a “quick and dirty” usability scale. Usability evaluation in industry.

[67] Chuttur MY. Overview of the technology acceptance model: origins, developments and future directions; Indiana University, USA. Sprouts Working Papers Inf Syst. 2009;9(37):1–23.

[68] Conradsson M, Lundin-Olsson L, Lindelof N, Littbrand H, Malmqvist L, Gustafson Y, Rosendahl E (2007). Berg Balance Scale: intrarater test-retest reliability among older people dependent in activities of daily living and living in residential care facilities. Phys Ther.

[69] Rubin DB (1987). Multiple imputation for nonresponse in surveys.

[70] Lamb SE, Jorstad-Stein EC, Hauer K, Becker C (2005). Development of a common outcome data set for fall injury prevention trials: the Prevention of Falls Network Europe Consensus. J Am Geriatr Soc.

[71] Rothman, K. J., and J. D. Boice, Jr. 1982. Epidemiologic analysis with a programmable calculator. Brookline, MA: epidemiology resources”. For the IRR estimation and “Greenland, S., and J. M. Robins. 1985. Estimation of a common effect parameter from sparse follow-up data. Biometrics 41: 55–68. 4005387

[72] Campbell AJ, Robertson MC (2003). Otago exercise programme to prevent falls in older people.

[73] Kyrdalen IL, Moen K, Røysland AS, Helbostad JL (2014). The Otago exercise program performed as group training versus home training in fall-prone older people: a randomized controlled trial. Physiother Res Int.

[74] Hunter RM, Baio G, Butt T, Morris S, Round J, Freemantle N (2015). An educational review of the statistical issues in analysing utility data for cost-utility analysis. PharmacoEconomics.

[75] White IR, Royston P, Wood AM (2011). Multiple imputation using chained equations: issues and guidance for practice. Stat Med.

[76] Faria R, Gomes M, Epstein D, White IR (2014). A guide to handling missing data in cost-effectiveness analysis conducted within randomised controlled trials. PharmacoEconomics.

[77] Manca A, Hawkins N, Sculpher MJ (2005). Estimating mean QALYs in trial-based cost-effectiveness analysis: the importance of controlling for baseline utility. Health Econ.

[78] National Institute for Health and Care Excellence (NICE) (2018). Guide to the process of technology appraisal.

[79] Bangor A, Kortum P, Miller J (2009). Determining what individual SUS scores mean: adding an adjective rating scale. J Usability Stud.

[80] Donoghue D, Stokes EK (2009). How much change is true change? The minimum detectable change of the Berg Balance Scale in elderly people. J Rehabil Med.

[81] Saso A, Moe-Nilssen R, Gunnes M, Askim T (2016). Responsiveness of the Berg Balance Scale in patients early after stroke. Physiother Theory Pract.

[82] Cook JA, Julious SA, Sones W, Rothwell JC, Ramsay CR, Hampson LV, Emsley R, Walters SJ, Hewitt C, Bland M, Fergusson DA, Berlin JA, Altman D, Vale LD (2018). DELTA^2^ guidance on choosing the target difference and undertaking and reporting the sample size calculation for a randomised controlled trial. Trials.

[83] Godi M, Franchignoni F, Caligari M, Giordano A, Turcato AM, Nardone A (2013). Comparison of reliability, validity, and responsiveness of the mini-BESTest and Berg Balance Scale in patients with balance disorders. Phys Ther.

[84] Picorelli AMA, Pereira LSM, Pereira DS, Felício D, Sherrington C (2014). Adherence to exercise programs for older people is influenced by program characteristics and personal factors: a systematic review. J Phys.

[85] Hawley-Hague H, Horne M, Skelton DA, Todd C (2016). Review of how we should define (and measure) adherence in studies examining older adults’ participation in exercise classes. BMJ Open.

[86] Moore G, Audrey S, Barker M, Bond L, Bonell C, Hardeman W, Moore L, O’Cathain A, Tinati T, Wight D, Baird J. Process evaluation of complex interventions: Medical Research Council guidance. BMJ. 2015, 2015:350–h1258. 10.1136/bmj.h1258 PMC4366184 25791983

[87] Agency for Healthcare Research and Quality. Reliability Testing of the AHRQ EPC Approach to Grading the Strength of Evidence in Comparative Effectiveness Reviews U.S. Department of Health and Human Services 540 Gaither Road Rockville, MD 20850. 2012. Available at www.ahrq.gov. Accessed 17 Aug 2019. 22764383

[88] Duckham RL, Masud T, Taylor R, Kendrick D, Carpenter H, Iliffe S, Morris R, Gage H, Skelton DA, Dinan-Young S, Brooke-Wavell K (2015). Randomised controlled trial of the effectiveness of community group and home-based falls prevention exercise programmes on bone health in older people: the ProAct65+ bone study. Age Ageing.

[89] Iliffe S, Kendrick D, Morris R, Skelton D, Gage H, Dinan S, Stevens Z, Pearl M, Masud T (2010). Multi-centre cluster randomised trial comparing a community group exercise programme with home based exercise with usual care for people aged 65 and over in primary care: protocol of the ProAct 65+ trial. Trials.

