# Supplementary material for: The effectiveness and cost-effectiveness of strength and balance Exergames to reduce falls risk for people aged 55 years and older in UK assisted living facilities: a multi-centre, cluster randomised controlled trial
Source: BMC Med. 2019 Feb 28;17:49. doi: 10.1186/s12916-019-1278-9 (PMC6394073; doi:10.1186/s12916-019-1278-9)
Supplement: Supplementary file 1 — Table S1. Exercise description and corresponding games. Figure S1. An example of a participant Exergame schedule. Table S2. Games and description of play. (DOCX 876 kb) [file 12916_2019_1278_MOESM1_ESM.docx]

**Additional file 1**

MIRA Exergames include 16 games implementing strength, balance, co-ordination and flexibility exercises. Several exercises can be performed for more than one game, and most of the games can be played by performing several different exercises. There is an option at the beginning of each game to watch detailed tutorial on the correct movements for the exercises and to understand how to play the game.

**Table S1. Exercise description and corresponding games**

| ILLUSTRATION | NAME AND DESCRIPTION | CORRESPONDING GAMES |
| --- | --- | --- |
| 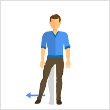 | **Side Taps**  The body should be held straight, with both legs held straight and on the ground. The legs should be apart from each other. The exercise involves extending each leg in turn to the side with the toes touching the ground, without making a full step. | Atlantis, Catch, Izzy the Bee, Move, Follow  Jugger, Firefly, Catch, Follow, Move |
| 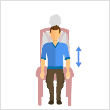 | **Sit to Stand**  The body should be sitting on a chair, with the feet on the ground. The exercise involves sitting tall near the front of the chair, followed by sitting back into the chair. This exercise targets lower limb strength and balance. | Powerhouse Bid, Atlantis, Izzy the Bee |
| 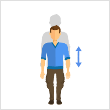 | **Squats**  The body should be straight, with both legs held straight and on the ground. The exercise involves bending the knees, lowering the torso, and then returning to the upright position. This exercise targets lower limb strength and balance. | Izzy the Bee, Atlantis, Catch, Move, Follow |
| 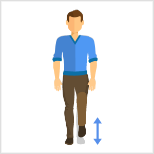 | **Hip Frontal Flexion**  The leg should be held straight and on the ground. The exercise involves raising the leg to the front, keeping it in a straight line with the body. This exercise targets flexibility, lower limb strength and balance. | Animals, Atlantis, Airplane, Catch, Follow, Colour Clouds, Move, Piano. |
| 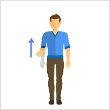 | **Elbow Flexion**  The arm should be kept close to the body. The exercise consists of raising the hand towards the chest, by bending the elbow. This exercise targets flexibility and strength. | Grab, Catch, Follow, Atlantis, Izzy the Bee, Move, Firefly,Piano, Jugger. |
| 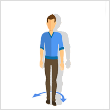 | **Full Body Turn**  The body should be held straight, with both legs held straight and on the ground. The exercise involves turning your entire body to the left and right, while making steps in place. This exercise targets balance and co-ordination. | Animals, Catch, Firefly, ColourClouds, Follow, Jugger, Basketball, Move. |
| 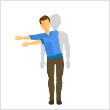 | **Functional Reach**  The body should be held straight. The exercise involves stretching the arms and reaching out from the torso, without stepping out of the initial position, in order to follow the objectives in the associated game. | Grab |
| 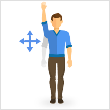 | **General – Shoulder**  The arm should be held straight. The exercise involves moving the arm, while keeping it straight, in order to follow the objectives in the associated game. This exercise targets flexibility, strength, coordination and function. | Atlantis, Catch, Firefly, Follow, Izzy the Bee, Jugger, Move |
| 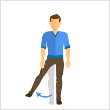 | **Hip Abduction**  The leg should be held straight and on the ground. The exercise involves raising the leg away from the side, keeping it in a straight line with the body. This exercise targets flexibility, lower limb strength, balance. | Atlantis, Catch, Izzy the Bee, Move, Follow  Fireflies, Jugger, Seasons. |
| 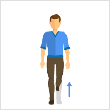 | **Knee Flexion**  The leg should be held straight and on the ground. The exercise involves lifting the leg by drawing the heel backwards, toward the bottom, but keeping the knee in line with the hip. This exercise targets flexibility, strength, and balance. | Grab, Catch, Follow, Atlantis, Izzy the Bee, Move, Firefly, Jugger. |
| 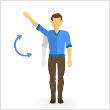 | **Shoulder Abduction**  The arm should be kept close to the body. The exercise consists of raising the arm away from the side, keeping it in a straight line with the body. This exercise targets flexibility and strength. | Atlantis, Follow, Catch, Izzy the Bee, Move  Catch, Firefly, Jugger, Follow, Memory scape, Move |
|  |  |  |

**Figure S1. An example of a participant Exergame schedule**

Exergame programmes are set by the therapist, and tailored according to the participant’s needs with the planned duration, rest time and number of Exergames. They are then saved in a Schedule, which can be replayed or adjusted as required.


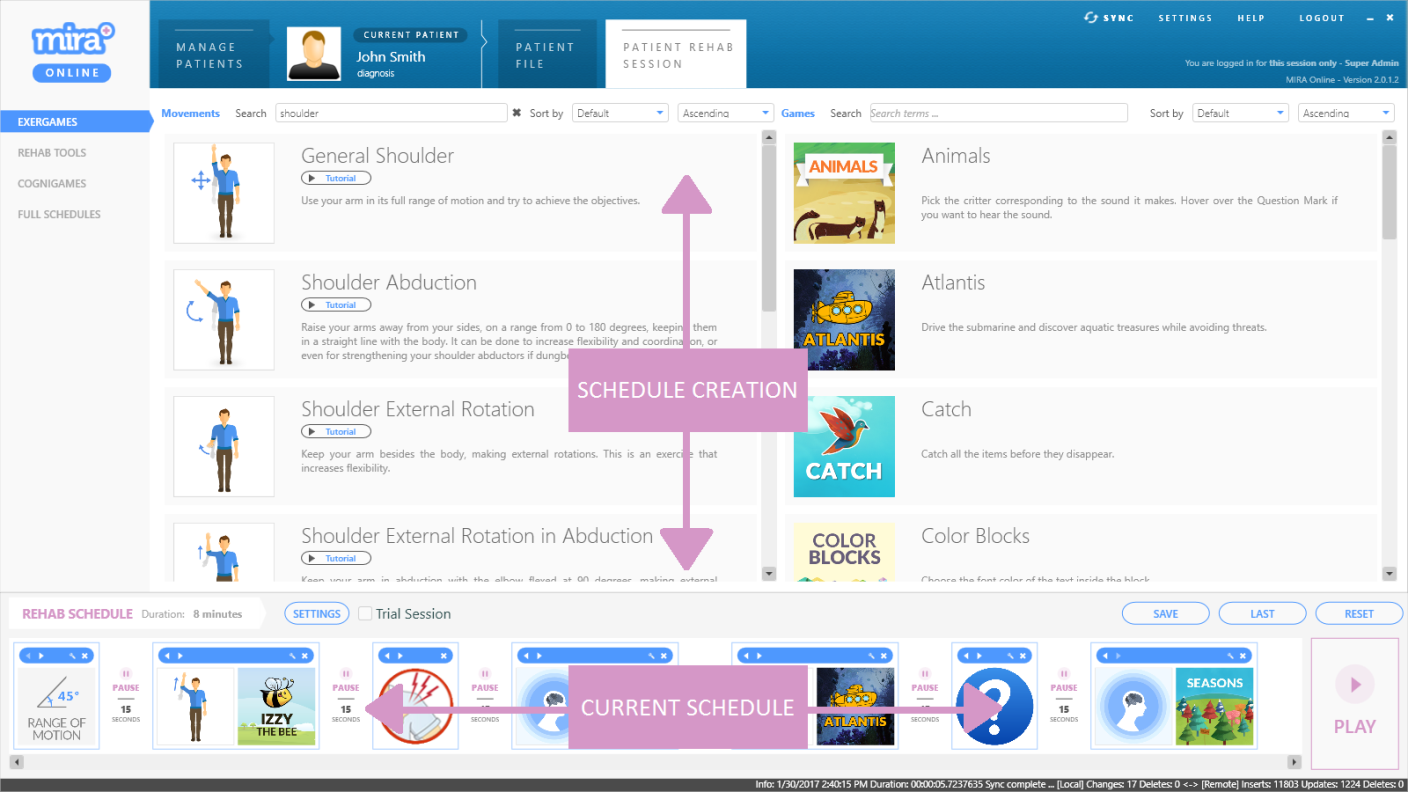


**Table S2. Games and description of play.**

| ILLUSTRATION | NAME AND DESCRIPTION OF GAME |
| --- | --- |
| 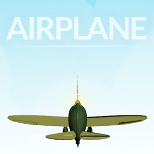 | **Airplane**  The player will have to pilot a plane, with the objective of shooting different targets that appear in the sky. |
| 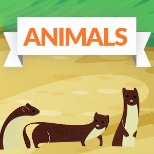 | **Animals**  The player's objective is to pair different animals to the noise they make. For each round, they will see two animals and hear a certain noise. Then, they will have to choose the animal that makes the noise they hear. |
| 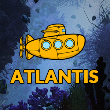 | **Atlantis**  The player will have to pilot a submarine through the depths of the ocean. The objective is to study certain artefacts that appear in the player's journey, while avoiding underwater mines or other perils that may cross their path. |
| 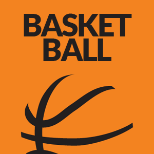 | **Basketball**  The player will have to beat basketballs that appear around them, while not letting them escape outside the screen. |
| 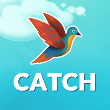 | **Catch**  The objective is to catch different objects that appear on the screen. They can move or stand still, but will always disappear in a few seconds, so the player will have to make sure to catch them while they can. |
| 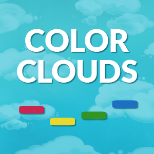 | **Colour Clouds**  The player will see two clouds on the screen, each with a word representing a colour written inside it. The player will have to answer if the colour of the text written on the right cloud is the same as the colour written on the left cloud. |
| 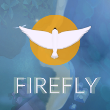 | **Firefly**  The objective is to fly a dove up in the sky. The player will have to collect fireflies that appear in their path and then guide them to safe havens to keep them out of peril. The player will have to make sure to avoid vultures or other dangers that may come their way. |
| 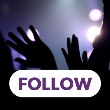 | **Follow**  The objective is to follow a coloured rectangle that moves randomly on the screen. The longer the player stays inside the rectangle, the higher the volume of the music inside the game will go. |
| 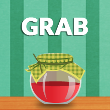 | **Grab**  The player will have to move different jars or other items in the pantry on different shelves. The player will have to make sure not to drop them, or they will break. |
| 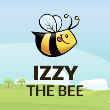 | **Izzy the Bee**  The player will have to help Izzy in her quest to make honey. The objective is to gather pollen and nectar from flowers and then deposit them to bee hives which will produce honey. The player will have to make sure to avoid bugs or other perils who will steal what they have gathered. |
| 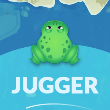 | **Jugger**  The player will have to help Jugger the frog jump from lily pad to lily pad, while avoiding falling in the water. |
| 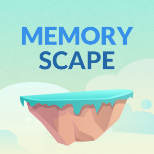 | **Memory Scape**  The player will see some cards arranged on the screen, each card having drawn a certain symbol. From the second turn on, the first card from the right will be turned with the symbol towards the player, while all the other cards will be turned upside down, with the symbol hidden. The player will have to answer if the symbol hidden under the first card on the left is the same as the visible symbol on the first card on the right. With every passing turn, all the cards will move with a position to the left. When the answer is wrong, the cards are reshuffled. |
| 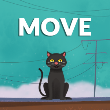 | **Move**  Before each game round, the player will see an image on the screen, with some missing items. After the image disappears, they will see each missing item at the beginning of a path. The player will have to move the item alongside the path, without skipping any sections, towards the end of the path. When the end is reached, the player will have to hold the item in place for a few seconds. Once all the items have been moved, the player will have completed the image with all the missing items and the game will go to the next round. |
| 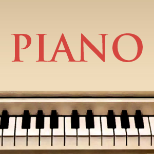 | **Piano**  The objective is to play some short tunes on a piano keyboard. For each game round, the player will first hear the tune they will have to play, and then, the keyboard will appear on the screen. From here on, the player will have to hit the highlighted keys to play the tune they heard at the beginning. |
| 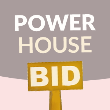 | **Powerhouse Bid**  The player will have to bid for certain objects in an auction house for antiquities. The player will have to make sure to outbid the other players for items they want to buy. |
| 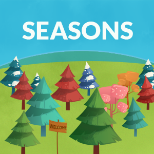 | **Seasons**  The player will see a landscape with some objects on the ground. Seasons will pass, and each passing season corresponds to a turn in the game, and for each turn, a new item will appear. For each turn, the player will have to choose an item which they haven't chosen already. After all the objects have appeared, the game will advance to the next round and start all over again. |
